# Covid-19-Studien im Vergleich: Aktives Sporttreiben und passive Bildschirmzeiten in der Pandemie und die Auswirkungen auf das körperliche und psychosomatische Wohlbefinden von Kindern und Jugendlichen in Deutschland und Europa

**DOI:** 10.1007/s43594-021-00043-8

**Published:** 2021-11-05

**Authors:** Roland Naul

**Affiliations:** Willibald Gebhardt-Institut e. V., Münster, Deutschland

Im Vierten Deutschen Kinder- und Jugendsportbericht (Breuer et al. 2020) gehen die Herausgeber*innen im Rahmen ihrer zusammenfassenden Kernaussagen am Rande auf die (möglichen) Auswirkungen der Corona-Pandemie ein. Für Deutschland verweisen sie auf eine erste Studie von Mutz und Gerke (2020) und auf zwei europäische Studien (Pietrobelli et al. 2020; Zenic et al. 2020) sowie eine US-amerikanische Studie (Dunton et al. 2020), ohne allerdings auf die Inhalte und vorliegenden Ergebnisse dieser Studien einzugehen. Die Herausgeber*innen formulieren „einige Annahmen“ (Breuer et al. 2020, S. 9), die als Auswirkungen der Corona-Pandemie für Kinder und Jugendliche vermutet werden: sportartspezifische Kompetenzen würden als Kohorteneffekt nur verzögert entwickelt werden; ein „Rückgang des Anteils sport- und bewegungsaktiver Kinder und Jugendlicher“ (Breuer et al. 2020, S. 9) wird vermutet und „die soziale Ungleichheit in Sport und Bewegung“ habe in ihrer Wirkung zugenommen (Breuer et al. 2020, S. 9). Zusammenfassend zu den Corona-Auswirkungen kommen die Autor*innen zu dem Schluss: „Vorhandene Probleme der Inaktivität von Kindern und Jugendlichen in Deutschland dürften zumindest vorübergehend zugenommen haben. Inwieweit dies motorische oder gesundheitliche Folgen hat, bleibt zu untersuchen“ (Breuer et al. 2020, S. 10).

Somit unterbleibt im Vierten Kinder- und Jugendsportbericht eine argumentative Auseinandersetzung zu den vermuteten Kohorteneffekten durch die Corona-Pandemie (vgl. auch: Kuhlmann 2021, S. 18). Eine Verschärfung der sozialen Ungleichheit im Zugang für Bewegung und Sport und der damit auch vermuteten Erhöhung der Inaktivität von Kindern und Jugendlichen in Corona-Zeiten besteht zu Recht. Das alles spiegelt, allerdings ohne solide Datenbasis, auch erwartbare Folgen und Einschränkungen für Kinder und Jugendliche im Ganztag wider, die wohl noch schärfer von diesen Auswirkungen betroffen sind nach mehr als einem Jahr Schließung der Schulen und Sportvereine. Denn dieses Klientel in der offenen Ganztagsschule verfügt weitgehend über keine weitreichende Bewegungs- und Sportsozialisation, kommt häufiger aus sozial schwachen, bildungsfernen und sportabstinenten Elternhäusern, wie wir aus den Studien zur Entwicklung von Ganztagsschulen der vergangenen Jahre und anderen Evaluationsstudien wissen (vgl. Naul und Neuber 2021). Umso mehr schmerzt der Verlust, dass über ein Jahr lang keine Bewegungs‑, Spiel- und Sportkurse im Ganztag und im Sportverein besucht werden konnten.

Die deutschlandweite, monatelange Schließung von Schulen und Sportvereinen in der Corona-Pandemie hat für alle Schüler*innen weitreichend ihre Inaktivität drastisch erhöht und durch das Homeschooling ihre Verweilzeiten an den Bildschirmen deutlich in die Höhe schnellen lassen. Wenn man einigen internationalen Studien zu Covid-19 Auswirkungen auf den Lebensstil von Kindern und Jugendlichen in Corona-Zeiten Glauben schenkt, haben sich auch weitere ungesunde Verhaltensweisen in der Ernährung eingestellt. Neben den physischen Einschränkungen haben mangelnder Schulbesuch und das Verbot, Freunde im Sportverein zu treffen, auch zu drastischen psychosozialen Folgen geführt, wie deutsche und europäische Studien zeigen. Um dazu insgesamt mehr Klarheit über vorliegende Forschungsergebnisse zu bekommen, werden im Folgenden einige deutsche und internationale Covid-19-Studien mit ihren Ergebnissen bilanziert.

## Deutsche Covid-19-Studien

**Figure 1:**
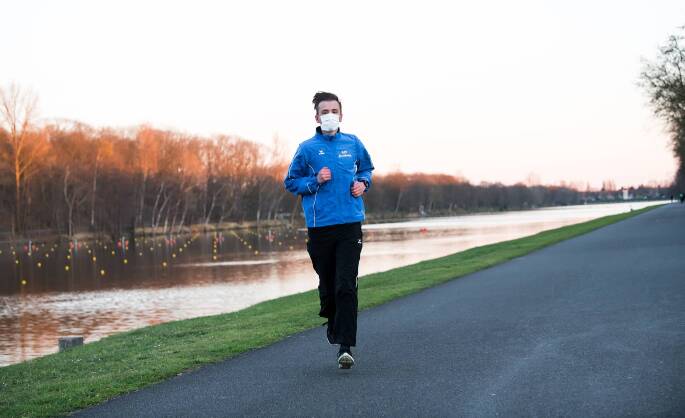

In einer frühen Studie von Mutz und Gerke (2020), die in einer Online-Befragung (*n* = 1001) deutsche Jugendliche und Erwachsene (14 bis 64 Jahre) in den ersten Wochen des Lockdowns Ende März/Anfang April 2020 durch Selbstauskunft befragt haben, wurden die Teilnehmer*innen nach ihrem Bewegungsverhalten vor der Pandemie und in den ersten zwei, drei Wochen zu Beginn der Pandemie gefragt, nachdem im Zuge des Lockdowns alle Schulen und Sportstätten geschlossen waren. Für das „leisure time sport & exercise“-Profil (LTSE) wurden vier Sample-Gruppen unterschieden: nicht engagiert in LTSE, reduziertes Engagement, aufrecht erhaltendes Engagement, erhöhtes Engagement. Waren vor der Pandemie 36 % der Befragten nicht engagiert, so stieg dieser Anteil auf 59,5 %. Insgesamt reduzierten 31 % ihr Engagement auf unterschiedlichen Aktivitätsgraden (Mutz und Gerke 2020, S. 305). Jene Gruppe, die zwei Stunden pro Woche aktiv waren (10,3 %), reduzierte ihren Anteil um 6,7 %, so dass in der Pandemie nur noch 3,6 % der Befragten diese zwei Stunden LTSE-aktiv waren. Rund 27 % aller Befragten hielten ihren Anteil während der Pandemie konstant, 6 % intensivierten ihren Anteil an LTSE, hauptsächlich durch weitere Outdoor-Aktivitäten (Laufen (Abb. 1), Radfahren) und durch Workouts zu Hause (Mutz und Gerke 2020, S. 309/310). Insgesamt führte die Pandemie schon in den ersten Wochen zu einem signifikanten Rückgang der Bewegungsaktivitäten, die in der jüngsten Teilnehmendengruppe (14 bis 29 Jahre) nicht so umfangreich waren wie bei den älteren Mitgliedern in dieser Studie.

Ähnliche Ergebnisse, was die Einschränkungen der Sport-Aktivitäten (SA) betrifft und deren geringere Auswirkungen für jüngere Altersgruppen in Deutschland, werden von Schmidt et al. (2020) berichtet. Die Arbeitsgruppe untersuchte auf der Grundlage von Daten der Motorik-Modul-Längsschnittstudie (MoMo) 2018 vor der Pandemie und einer Befragung mit dem MoMo-Fragebogen (PAQ) 1711 Kinder und Jugendliche im Alter von 4 bis 17 Jahren, unterteilt in 4‑ bis 5‑, 6‑ bis 10-, 11- bis 13- und 14- bis 17-Jährige in den ersten Wochen des Lockdowns. Dabei standen drei zentrale Fragestellungen im Vordergrund der Untersuchung: (1) inwieweit wurde von der Untersuchungsgruppe ihr vormals aktives, organisiertes Sporttreiben vor der Pandemie während der frühen Wochen in der Pandemie (Datenerhebung im April 2020) in selbst-organisiertes Sporttreiben umgewandelt, (2) inwieweit betraf die Pandemie die „habitual physical activities“ (HPA, draußen spielen, Wandern, Fahrradfahren, Gartenarbeit und Hausarbeit) vor und in der Pandemie und (3) inwieweit haben sich die Zeiten für Sportaktivitäten, HPA und Bildschirmzeiten in den Pandemiewochen verändert?

**Figure 2:**
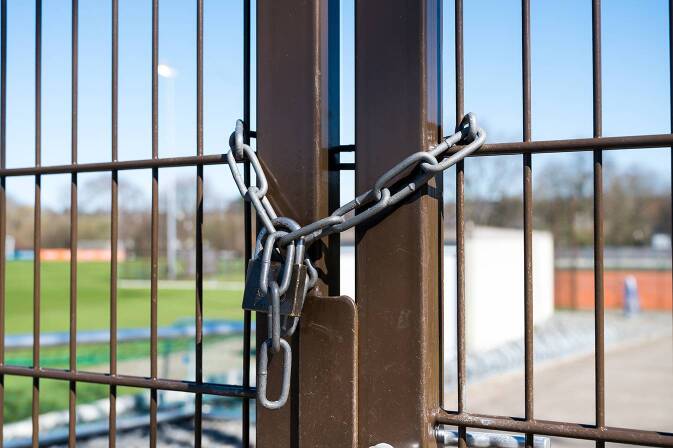

Da in den frühen Wochen der Pandemie alle Schulen und Sportvereine geschlossen wurden, gab es für die Kinder und Jugendlichen keine organisierten Sportangebote (SA) mehr (0,0 min am Tag, Abb. 2). Vor der Pandemie lautete der Durchschnittswert für das Gesamtsample 28,5 min SA pro Tag mit dem höchsten Tageswert von 35,8 min für die Altersgruppe der 11- bis 13-Jährigen. Der Tageswert für nicht- beziehungsweise selbstorganisiertes Sporttreiben lag vor der Pandemie für das Gesamtsample bei 6,6 min pro Tag, am höchsten mit 12,0 min bei der Altersgruppe der 14- bis 17-Jährigen (Schmidt et al. 2020, S. 4).

Während der Pandemiewochen wechselten mehr als 60 % der Aktiven aus ihrem organisierten Sportaktivitäten in eigene, nicht-organisierte Sportformen, wobei Wandern und Radfahren stabil blieben. Im Durchschnitt erhöhte sich das tägliche Minutenvolumen in der Pandemie für selbst-organisiertes Sporttreiben des Gesamtsamples von 6,6 min auf 24,3 min mit der höchsten Steigerungsrate von 6,7 min auf 29,7 min für die 11- bis 13-Jährigen. Ebenso ermittelten die Autor*innen dieser Studie Veränderungsprozesse für den Index aller HPA-Aktivitäten vor der Pandemie (pre) und in der Pandemie (pri). Hier kommt es bei HPA für das Gesamtsample zu einer Steigerung von 36,2 min in der Pandemie (von 107,3 min auf 143,5 min) mit dem höchsten Steigerungswert für die 4‑ bis 5‑Jährigen (plus 57,7 min) (Schmidt et al. 2020, S. 5). Beide Steigerungswerte in der Pandemie für „non-organized sports“ und „habitual physical actvities“ werden addiert und mit den Vorpandemiewerten für das organsierte Sporttreiben verglichen. So kommen die Autor*innen zu dem Ergebnis, dass sich in der Pandemie der Gesamtaktivitätsindex um 11,1 % angeblich erhöht habe, bei einem gleichzeitigen Verlust von insgesamt 10,8 min pro Tag für den Sport. Wegen der sehr unterschiedlichen körperlichen Belastungswerte für das organisierte und selbst-organisierte Sporttreiben sowie für Formen von HPA (mit draußen spielen, Gartenarbeit und Hausarbeit) täuschen diese aufaddierten Minutenergebnisse in der Pandemie eine Stabilität beziehungsweise sogar eine positive Veränderung im Aktivitätsindex vor, die bei einem genauen Datenvergleich zwischen den Werten für das vor-pandemische organsierte Sportreiben mit den Werten für das selbst-organisierte Sporttreiben in der Pandemie von uns nicht bestätigt werden kann. Den insgesamt 28,5 min pro Tag für das vor-pandemische, organisierte Sporttreiben (Gesamtsample) stehen 24,3 min des selbstorganisierten Sporttreibens in der Pandemie gegenüber, wobei hier 6,6 min noch abgezogen werden müssten (= 17,7 min), denn dieser Wert entsprach dem Minutenvolumen schon in vor-pandemischen Zeiten. Insofern haben die Autor*innen recht, wenn sie den Totalverlust des Sporttreibens nach Abzug des Kompensationswertes für das selbst-organsierte Sporttreiben auf minus 10,8 min pro Tag kalkulieren (vgl. Schmidt et al. 2020, S. 6).

Klar und eindeutig sind indessen die Veränderungswerte für die Bildschirmzeiten. Die täglichen Zeiten haben sich signifikant gegenüber der Zeit vor der Pandemie um rund eine Stunde pro Tag erhöht (von 133,3 min auf 194,5 min), mit der höchsten Steigerungsrate für die 14- bis 17-Jährigen (67,8 min) (Schmidt et al. 2020, S. 6). Selbst wenn man, wie die Autor*innen, die zusätzlichen Minutenvolumina für HPA in der Pandemie zum Wert des reduzierten Gesamtwerts für das Sporttreiben in der Pandemie aufaddiert, so bleibt zum Schluss das Ergebnis: Die Sitzstunden an Bildschirmgeräten pro Woche übertreffen den Umfang an Bewegungsaktivitäten bei weitem, eben deutlich mehr in der Pandemie als in Zeiten davor.

**Figure 3:**
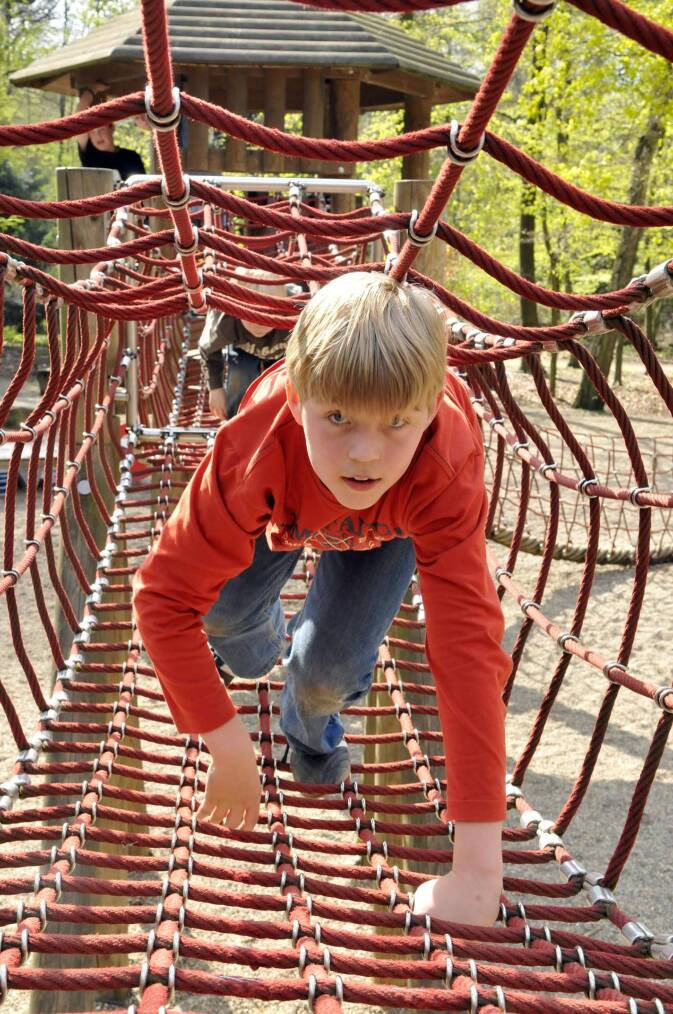

Eine zweite Studie von Schmidt et al. (2021) untersucht ihre Kohorte von 4‑ bis 17-Jährigen knapp sechs Monate später in der zweiten Covid-19 Lockdown-Welle (*n* = 1322) im Zeitraum von November 2020 bis Februar 2021. Für die Wintermonate des zweiten Lockdowns ergibt sich ein ganz anderes Datenbild als vorher im ersten Lockdown. Das bestätigt weitgehend die Datenanalyse aus der Studie 2020, was den Rückgang der organsierten Sportaktivitäten betrifft und deren nicht vorhandene Kompensation durch das eigene, unorganisierte Sporttreiben. Nur mit dem methodisch zu kritisierenden zusätzlichen Heranziehen von Minutenvolumina für körperliche Alltagsaktivitäten (HPA) wurde in der Studie 2020 der Eindruck erweckt, das Bewegungsprofil habe sich im ersten Lockdown insgesamt erhöht gegenüber den Minutenwerten für das organisierte Sporttreiben vor der Pandemie. In der zweiten Studie mit neuen Daten wird Folgendes deutlich: Die Teilnahme am organisierten Sport ist von 0 min (runter von 26,3 min) im ersten Lockdown unwesentlich auf 3,7 min im zweiten Lockdown angestiegen; stieg hingegen im ersten Lockdown das Minutenvolumen für das unorganisierte Sporttreiben auf 23,9 min (rauf von 6,2 min vor der Pandemie), so gibt es im zweiten Lockdown einen gravierenden Rückgang auf nur 9,9 min (Schmidt et al. 2021, S. 10). Im Längsschnitt der drei Messzeitpunkte (vor Corona, erster Lockdown, zweiter Lockdown) ergibt sich also ein kontinuierlicher Rückgang für das Sporttreiben insgesamt von ursprünglich 32,5 min pro Tag über 23,9 min (erster Lockdown) auf 13,6 min (zweiter Lockdown), also ein Gesamtrückgang von fast 20 min pro Tag (Schmidt et al. 2021, S. 10). Auch für wesentliche Formen der körperlichen Alltagsaktivitäten (HPA) muss dieser Gesamttrend berichtet werden: Spielen im Freien (Abb. 3) (59,1 min vor dem Lockdown) soll sich im ersten Lockdown auf 81,7 min erhöht haben, umfasste aber im zweiten Lockdown nur noch 22,4 min und lag damit auch deutlich unter dem Wert vor der Pandemie (Schmidt et al. 2021, S. 11). Eine ähnlich rückläufige Tendenz gegenüber den Werten vor der Pandemie und den Werten für den ersten Lockdown, wenngleich nicht so hoch, ergab sich für das tägliche Radfahren (8,1 vs. 11,3 vs. 5,1 min) im zweiten Lockdown.

Bei der Mediennutzung haben vor der Pandemie 61,9 % der Kinder und Jugendlichen den Richtwert von maximal zwei Stunden Bildschirmzeit in der Freizeit nicht überschritten. Im ersten Lockdown waren dies nur noch 38,6 % und im zweiten Lockdown sank dieser Wert noch einmal auf 30,6 %. Hatte sich in der ersten Studie von 2020 das Minutenvolumen der Bildschirmzeit um rund eine Stunde erhöht (von 130,8 min auf 191,2 min), so beträgt dieser Wert nun in der zweiten Lockdown-Studie 227,5 min, also fast 100 min mehr als vor der Corona-Pandemie (Schmidt et al. 2021, S. 12).

Alles in allem kann für die zweite Lockdown-Studie von Schmidt et al. (2021) festgehalten werden: Es gibt einen deutlichen Rückgang für sportliche Aktivitäten von 32,5 min pro Tag vor der Pandemie auf 13,6 min im zweiten Lockdown, also rund 20 min weniger, während der Medienkonsum gleichzeitig pro Tag um knapp 100 min zugenommen hat. Bemerkenswert ist auch, dass nach der Selbsteinschätzung der Untersuchungsgruppe 48 % meinen, ihre Fitness habe sich verschlechtert und 38 % eine Gewichtszunahme „fühlen“ (Schmidt et al. 2021, S. 13).

Eine andere umfangreiche Studie mit Kindern zwischen 4 und 10 Jahren (*n* = 514) und Jugendlichen zwischen 11 und 17 Jahren (*n* = 750) wurde auf der Grundlage der dritten Welle der MoMo-Studie durchgeführt (Wunsch et al. 2021). Dabei wurde untersucht, inwieweit die Zusammenhänge zwischen körperlicher Aktivität, Bildschirmzeiten und der „Health Related Quality of Life“ (HRQoL) in Vor-Corona Zeiten (2018) eine Voraussage erlauben für den Zusammenhang dieser Indikatoren in Corona-Zeiten (Ende April/Anfang Mai 2020). Die Autor*innen konnten von ihren vier Hypothesen nur eine Hypothese bestätigen. „The exploratory analysis revealed a positive effect of HRQoL pre-COVID-19 on PA within-COVID-19, especially in younger children and in females“ (Wunsch et al. 2021, S. 9). Dieses Ergebnis stützt andere Untersuchungen, die einen positiven Einfluss von HRQoL auf ihr weiteres Sporttreiben festgestellt haben. Was in dieser Studie mehr überrascht, sind jedoch die vorgestellten Ergebnisse zum Umfang der körperlichen Aktivität der beiden Sample-Gruppen vor der Corona-Zeit und in den frühen Wochen 2020 in der Corona-Zeit, als alle Schulformen geschlossen waren, Ganztagsangebote und auch der Besuch von Sportkursen in den Vereinen wegfielen. So wird für die Altersgruppe der 4‑ bis 10-Jährigen berichtet, ihr Anteil, bei Jungen und Mädchen, an wöchentlichen Tagen, an denen sie die Norm von 60 min Bewegungszeit pro Tag erreicht haben, habe sich bei Jungen von 4,74 auf 5,39 Tage und bei Mädchen dieser Altersgruppe von 4,62 auf 5,27 Tage in der Corona-Pandemie sogar erhöht! Das Gleiche wird für die Altersgruppe der 11- bis 17-Jährigen mitgeteilt (Jungen von 3,90 Tage auf 4,08 Tage, Mädchen von 3,55 Tage auf 3,96 Tage) (Wunsch et al. 2021, S. 8). Wegen der Schließung von Schulen und Sportvereinen haben sich die körperlichen Bewegungszeiten in der Freizeit zu Hause infolge von Corona erhöht.

Diese Ergebnisse (Schmidt et al. 2020; Wunsch et al. 2021) überraschen und stehen im Gegensatz zu der Mehrzahl anderer Studien, wie noch zu zeigen sein wird. Allerdings müssen wir hier vor einer vorschnellen Schlussfolgerung warnen, Corona habe sich insgesamt positiv auf den Umfang des Bewegungsverhaltens ausgewirkt. Für Bewegungsaktivitäten in Haus und Hof und in der schul- und vereinsfreien Corona-Freizeit mögen diese Werte leicht gestiegen sein. Aber diesem leichten bis moderaten Anstieg steht pro Woche der Verlust von zwei bis drei Stunden Schulsport, der weitere Verlust von durchschnittlich zwei Sportkursen für Ganztagsschüler und weitere zwei bis vier Stunden Sporttreiben für jugendliche Vereinsmitglieder gegenüber.

Summa summarum kann also hier aufaddiert nur von einem insgesamt erheblichen Rückgang der Sportaktivitäten geredet werden, der in keiner Weise von dem leichten Anstieg der Bewegungszeiten zu Corona-Freizeiten kompensiert wird. Mehr im Einklang mit anderen Studien stehen bei Wunsch et al. (2021) ihre Minutenvolumina für Bildschirmzeiten pro Tag für die beiden Untersuchungsgruppen. Für die 4‑ bis 10-jährigen Jungen verdoppeln sich fast diese Bildschirmzeiten in der Corona-Zeit (76,65 auf 143,73 min). Bei den Mädchen in dieser Altersgruppe ist diese Steigerung nicht ganz so groß (74,61 min auf 124,86 min). Bei den älteren Jugendlichen ist der Steigerungswert geringer, dafür aber ist das Gesamtminutenvolumen in Corona-Zeiten pro Tag deutlich höher geworden (Jungen 233,10 vs. 299,42 min, Mädchen 186,13 vs. 250,25 min) (Schmidt et al. 2021, S. 8).

Neben deutlichen Veränderungen im sportlichen und sonstigen Bewegungsverhalten, die durch die Corona-Pandemie verursacht worden sind, gibt es auch gravierende Auswirkungen im psychosomatischen Verhaltensbereich bei Kindern und Jugendlichen. So haben Ravens-Sieberer et al. (2021a) nach einer Studie über die Auswirkungen der ersten Covid-19 Welle (COPSY I) ein halbes Jahr später weitere Ergebnisse aus der zweiten Welle erhoben (COPSY II) und somit längsschnittliche Vergleiche zwischen dem psychosomatischen Verhaltensprofil von Kindern und Jugendlichen vor der Pandemie (t1), in der ersten Welle der Pandemie (t2) und nach einem weiteren Halbjahr aus der zweiten Welle (t3) vorgelegt (Ravens-Sieberer et al. 2021b). Es wurden zwei Altersgruppen untersucht, die 7‑ bis 10-Jährigen (t2 *n* = 546; t3 *n* = 503) und die 11- bis 17-Jährigen (t2 *n* = 1040; t3 *n* = 1077). Das Durchschnittsalter der Samples betrug 12,25 Jahre (t2) und 12,75 Jahre (t3). Es kamen verschiedene Messinstrumente zum Einsatz (KIDscreen, SDQ, HRQol-Fragebogen u. a.). Zu rund 85 % blieb das Sample zwischen t2 und t3 stabil, das heißt, wir haben es weitgehend mit einem echten Längsschnitt zu tun. Auffallend ist bei den Ergebnissen, dass ein niedriger Wert im „Health Related Quality of Life“ (HRQoL) bei Kindern und Jugendlichen während der Pandemie deutlich zugenommen hat (von 15,3 % vor der Pandemie auf 40,2 % in der ersten Welle und weiter auf 47,7 % in der zweiten Welle). Analog hat sich der Wert für einen hohen HRQoL deutlich reduziert (von 16,6 % vor der Pandemie auf 5,8 % in der ersten Welle und 5,7 % in der zweiten Welle) (Ravens-Sieberer et al. 2021b, S. 23). Es liegen also reziproke Entwicklungen vor: der Anteil von einem niedrigen HRQoL hat sich verdreifacht, während der Anteil an einem hohen HQRoL auf ein Drittel geschrumpft ist. Ebenso haben sich die Klagen über Magenschmerzen (21,3 % vs. 30,5 % vs. 36,4 %) und Kopfschmerzen (28,3 % vs. 40,5 % vs. 46,4 %) in den zwei Wellen der Pandemie deutlich verstärkt (Ravens-Sieberer et al. 2021b, S. 24). Weiterhin treten mehr Klagen über Angstzustände, Depressionen und psychosomatisches Unwohlsein im Zuge der Pandemie bei den Kindern und Jugendlichen deutlich hervor.

## Europäische Covid-19-Studien

Ergänzt und erweitert werden diese Auswirkungen der Pandemie auf die Gesundheit, das Bewegungsverhalten, die motorische Entwicklung und das psychosomatische Wohlbefinden durch Erkenntnisse von Studien in anderen europäischen Ländern, in denen der Lockdown für Kinder und Jugendliche noch drastischer und umfangreicher war (zum Beispiel in Italien und Spanien).

So berichten beispielsweise in der Studie von Orgiles et al. (2020) 1143 Eltern aus 94 Städten Italiens und 87 Städten Spaniens über die Auswirkungen der Pandemie auf ihre Kinder im Alter von 3 bis 18 Jahren. Das Bewegungsveralten hatte sich über alle Aktivitätsgrade hinweg reduziert. Lag der Anteil der Kinder und Jugendlichen, die sich täglich rund 30 min bewegen, vor der Pandemie bei 13,6 %, so stieg der Wert auf 55,6 %, gleichzeitig sank aber der Wert für jene Gruppe, die sich täglich zwischen 60 und 90 min bewegen, von 28 % auf 9,3 % (Orgiles et al. 2020, S. 10). Ebenso sank der Anteil der Kinder und Jugendlichen, die täglich weniger als 30 min Medienzeit hatten, von 22,1 % auf nur 3,4 %. Dafür stieg der Anteil bei der Gruppe, die täglich 120 bis 180 min Bildschirmzeit hatten, von 5,5 % auf knapp 30 % (Orgiles et al. 2020, S. 10). Insgesamt klagten rund 86 % der befragten Eltern, dass 76 % ihrer Kinder an Kontaktmangel leiden, 52 % Langeweile haben, 31 % Einsamkeit empfinden und 30 % sich Sorgen machen (Orgiles et al. 2020, S. 6).

Im Prinzip werden diese Entwicklungen auch in der kleinen Studie aus Verona (Italien) bestätigt, in der 41 übergewichtige Kinder in ihrem Verhaltensprofil vor (Mai/Juni 2019) und in der Pandemie (März/April 2020) untersucht wurden (Pietrobelli et al. 2020). Nach ihrer Studie reduzierten sich die Sportstunden von 3,60 h auf 1,29 h, während die Bildschirmzeiten von 2,76 h sich drastisch erhöhten auf 7,61 h (Pietrobelli et al. 2020, S. 1384). Auffallend war auch das Ernährungsverhalten mit signifikanten Veränderungen (Pietrobelli et al. 2020, S. 1384). Der Konsum von Kartoffelchips und Süßgetränken hatte sich mehr als verdoppelt im Vergleich zu der Vor-Corona-Zeit.

Inwieweit hier und in anderen Studien der europaweite Rückgang von Bewegungszeiten und der gleichzeitige deutliche Anstieg von Bildschirmzeiten Folgen haben für motorische Entwicklung und Fitness von Kindern und Jugendlichen, können dem jährlichen Monitoring von Kindern und Jugend lichen in slowenischen Schulen entnommen werden. So berichtet Starc (2020) nach Messungen der 7‑ bis 15-jährigen Alterskohorte in über 100 Schulen, dass in der Pandemie (Juni 2020) im Vergleich zur altersgleichen Kohorte des Jahres 2019, 69,5 % der Mädchen und 67,8 % der Jungen über den Verlust ihrer „physical fitness“ klagten (Starc 2020, S. 3). 56,8 % der Mädchen und 58,4 % der Jungen berichteten über eine Gewichtszunahme (Starc 2020, S. 4). In den Physical-Fitness-Tests gab es im Alterskohortenvergleich zwischen 2019 und 2020 ein signifikant schlechteres Ergebnis für die aerobe Ausdauer und die Koordination, besonders in den Altersgruppen der 7‑ bis 9‑jährigen Jungen und Mädchen (Starc 2020, S. 5).

Fasst man diese Ergebnisse aus deutschen und europäischen Studien über die Auswirkungen von Covid-19 auf das Bewegungsverhalten und die gesundheitliche Entwicklung von Kindern und Jugendlichen zusammen, so dürften die ermittelten Folgen für Kinder und Jugendliche aus unseren Ganztagsschulen nicht besser sein. Im Gegenteil: die bekannten Sozialindikatoren (Bildungsstand, Einkommen und Sportaffinität der Eltern) sind im Durchschnitt eher geringer als für die Eltern anderer Schüler*innenschaften aus der Halbtagsschule. Die größten Verlierer*innen im Sportverhalten, in der körperlichen und sportlichen Entwicklung und in der deutlichen Erhöhung eines inaktiven Lebensstils sind in Corona-Zeiten die Ganztagsschüler. Ihnen fehlt gleich dreifach Bewegung, Spiel und Sport: als Sportunterricht in der Schule, als Sportkurse am Nachmittag in der Ganztagsschule und als Spiel und Sport im Sportverein.

## Ausblick

**Figure 4:**
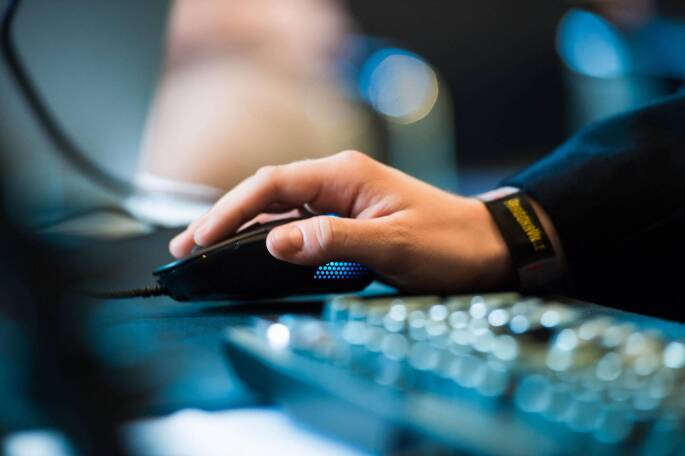

Aus deutschen und europäischen Studien über die Auswirkungen der Covid-19-Pandemie kann als eine gemeinsame Quintessenz ein drastischer Rückgang für das aktive Sporttreiben und sonstige Bewegungsverhalten in der Freizeit verzeichnet werden. Parallel dazu steigt ein passiver Lebensstil mit deutlich mehr Bildschirmzeiten (Abb. 4) pro Tag. Während teilweise die Sportaktivitäten auf die Hälfte bis ein Drittel des Wertes vor der Pandemie sinken, verdoppeln beziehungsweise verdreifachen sich die inaktiven Sitzzeiten vor den Bildschirmgeräten, nicht nur in Deutschland, sondern auch in anderen Mitgliedsländern der EU. Kinder und Jugendliche klagten deutlich öfter über psychosomatische Beschwerden in Corona-Zeiten als vor der Pandemie. Erste Monitoring-Ergebnisse im europäischen Ausland belegen einen Rückgang in der Entwicklung basismotorischer Kompetenzen und zeigen „gefühlte“ Gewichtszunahmen, die aus Elternberichten mit einem erhöhten Konsum von Snack-Food, Süßgetränken und Süßigkeiten einhergehen. Diese Tendenzen (Rückgang der Sportaktivitäten und Anstieg der Bildschirmzeiten) werden weitgehend auch in Studien aus den USA (Dunton et al. 2020) und China (Xiang et al. 2020) bestätigt.

Vergleichbare und erweiterte psychosoziale Auswirkungen der Covid-19-Pandemie neben den vorliegenden Ergebnissen von Ravens-Sieberer et al. (2021a, b) aus Deutschland, Orgiles et al. (2020) und Pietrobelli et al. (2020) aus Italien und Spanien werden vor allem aus Indien und anderen südostasiatischen Staaten gemeldet, in denen die Parameter Sporttreiben und Medienkonsum im Rahmen von Covid-Studien keine Bedeutung haben (Banerjal et al. 2020). Kinder und Jugendliche klagen dort auch deutlich öfter über psychosomatische Beschwerden in Corona-Zeiten als vor der Pandemie.

Während zahlreiche Ergebnisse zu den Corona-Folgen für das aktive Sporttreiben und den passiven Medienkonsum vorliegen, wissen wir indessen zu wenig, was deren mögliche Wirkungen für die Entwicklung des körperlich-gesundheitlichen und motorischen Aufwachsens bedeuten. Tendenziell, wie in der Studie von Starc (2020), wird in Slowenien über alters- und geschlechtsbezogene Rückgänge in der basismotorischen und Physcial-Fitness-Entwicklung in der Altersgruppe der 6‑ bis 12-Jährigen berichtet. Bisher liegt zu diesem Thema keine deutsche Monitoring-Studie vor, und es sind solche Ergebnisse auch europaweit rar. Deshalb ist es überfällig und naheliegend, eine solche Studie über die körperlichen, gesundheitlichen und vor allem motorischen Auswirkungen der Corona-Pandemie auf die Entwicklung der Corona-Kohorte des Jahres 2020/21 anzufertigen, auf deutscher und europäischer Ebene. Welche Auswirkungen hat zum Beispiel die fehlende Bewegungsförderung im zweiten Kita-Jahr bei Eintritt in das dritte Kita-Jahr? Was können die Kinder, die ihren Lockdown zum Beispiel im dritten Kita-Jahr hatten, nun basismotorisch bei Schuleintritt in die erste Klasse? Wie sieht es bei den Kindern aus, die jetzt ins zweite Schuljahr kommen, ohne jeglichen Sportunterricht in der ersten Schulklasse? Hier müsste in einem Forschungsverbund dringend diesen Fragen nachgegangen werden, um aktuell neue Förderakzente in Kita und Grundschule zu setzen. Im Prinzip gilt das auch für die vormaligen Viertklässler, die jetzt mit dem fünften Schuljahr in das weiterführende Schulsystem eintreten.

## References

[CR1] Banerjal D, Vaishnav M, Sathyanarayana TS, Raiju MSVK, Dalal PK, Javed A, Saha G, Mishra K, Kumar V, Jagiwala M (2020). Impact of the COVID-19 pandemic on psychosocial health and well-being in South Asian (World Psychiatric Association, zone 16) countries: A systematic and advocacy review from India. Indian Journal of Psychiatry.

[CR2] Breuer C, Joisten C, Schmidt W (2020). Vierter Deutscher Kinder und Jugendsportbericht.

[CR3] Dunton GF, Do B, Wang SD (2020). Early effects of the COVID-19 pandemic on physical activity and sedentary behavior in children living in the U.S. BMC Public Health.

[CR4] Kuhlmann D (2021). Was wissen wir über den Kinder- und Jugendsport?. Forum Kinder- und Jugendsport.

[CR5] Mutz M, Gerke M (2020). Sport and exercise in times of self-quarantine: how Germany changed their behaviour at the beginning of the COVID-19 pandemic. International Review of the Sociology of Sport.

[CR6] Naul R, Neuber N, Neuber N (2021). Sport im Ganztag – Zwischenbilanz und Perspektiven. Kinder- und Jugendsportforschung in Deutschland – Bilanz und Perspektive.

[CR7] Orgiles M, Delvecchio E, Mazzeschi C, Espada JP (2020). Immediate psychological effects of COVID-19 quarantine in Youth from Italy and Spain. Frontiers in Psychology.

[CR8] Pietrobelli A, Pecorano L, Ferruzzi A, Heo M, Faith M, Zoller T, Antoniazzi F, Piacentini G, Fearnbach N, Heymsfield SB (2020). Effects of COVID-19 Lockdown on lifestyle behaviors in children with obesity living in Verona, Italy: a longitudinal study. Obesity.

[CR10] Ravens-Sieberer U, Kaman A, Erhart M, Otto C, Devine J, Löffler C, Hurrelmann K (2021). Quality of life and mental health in children and adolescents during the first year of the COVID-19 pandemic: results of a two-wave nationwide population-based study.

[CR9] Ravens-Sieberer U, Kaman A, Erhart M, Devine J, Schlack R, Otto C (2021). Impact of the COVID-19 pandemic on quality of life and mental health in children and adolescents in Germany. European Child & Adolescents Psychiatry.

[CR11] Schmidt SCE, Anedda B, Burchatz A, Eichsteller A, Kolb S, Nigg C, Niessner C, Oriwol D, Worth A, Woll A (2020). Physical activity and screen time of children and adolescents before and during the COVID-19 lockdown in Germany: a natural experiment. Scientific Reports.

[CR12] Schmidt SCE, Burchatz A, Kolb S, Niessner C, Oriwol D, Hanssen-Doose A, Wortth A, Woll A (2021). Zur Situation der körperlich-sportlichen Aktivität von Kindern und Jugendlichen während der COVID-19 Pandemie in Deutschland.

[CR13] Starc G (2020). Physical fitness of Slovenian children after the COVID-19 lockdown.

[CR14] Wunsch K, Nigg C, Niessner C, Schmidt SCE, Oriwol D, Hanssen-Doose A, Buchartz A (2021). The impact of Covid 19 on the Interrelation of physical activity, screen time and health-related Quality of Life in children and adolescents in Germany: results of Motorik-Modul Study. Children.

[CR15] Xiang M, Zhang Z, Kuwahara K (2020). Impact of COVID-19 pandemic on children and adolescents’ lifestyle behaviour larger than expected. Progress in Cardiovascular Disease.

[CR16] Zenic N, Taiar R, Gilic B, Blazevic M, Maric D, Pojskic H, Sekulic D (2020). Levels and changes of physical activity in adolescents during the COVID-19 Pandemic: Contextualizing urban vs. rural living environment. Applied Sciences.

